# Atrial arrhythmias related to antitumor treatment in patients with coronary heart disease and malignant tumors

**DOI:** 10.3389/fonc.2025.1676263

**Published:** 2025-12-10

**Authors:** Beibei Zhang, Lei Zhang, Yili Ren, Chenkai Xu

**Affiliations:** 1 Department of Oncology, Zhejiang Hospital, Hangzhou, Zhejiang, China; 2 Department of hepatology I, Affiliated Hospital of Shaoxing University Department of Infectious Diseases, Shaoxing, Zhejiang, China; 3 Department of Infectious Diseases, Affiliated Hospital of Shaoxing University Department of Infectious Diseases, Shaoxing, Zhejiang, China; 4 Affiliated Hospital of Shaoxing University Geriatrics II, Shaoxing, Zhejiang, China; 5 Department of Cardiology, Zhejiang Hospital, Hangzhou, Zhejiang, China

**Keywords:** atrial arrhythmias, coronary heart disease, antitumor treatment, risk factors, cancer

## Abstract

**Aims:**

This study investigated the occurrence of arrhythmias in patients with malignant tumors and coronary heart disease (CHD) during antitumor drug treatment.

**Methods:**

109 patients with malignant tumors undergoing antitumor treatment at Zhejiang Hospital from January 2023 to January 2024 were analyzed. All patients underwent 12-lead ECG before and after each chemotherapy cycle. The primary endpoint was the occurrence of new arrhythmias recorded through ECG. Univariate and multivariate logistic regression analysis were performed to recognize the risk factors.

**Results:**

Atrial arrhythmias occurred in 13 cases (11.93%). The incidence of atrial arrhythmias was significantly higher in the CHD group compared to the non-CHD group (27.27% vs 1.54%; P < 0.001). Multivariate logistic regression analysis indicated that smoking (P = 0.009), ischemic heart disease (P = 0.006), and left ventricular ejection fraction < 50% (P = 0.014) increased the risk of developing arrhythmias after antitumor treatment.

**Conclusion:**

Atrial arrhythmias are more common in cancer patients with CHD when undergoing antitumor treatment. A history of smoking, ischemic heart disease, and reduced left ventricular ejection fraction increases the risk of atrial arrhythmias after antitumor therapy.

## Introduction

Coronary heart disease (CHD) is one of the leading causes of death worldwide, with mortality rate increasing from 27.3% to 31.4% in the past few decades (1). CHD refers to diseases caused by atherosclerotic plaque formation in the coronary arteries, leading to narrowing of the lumen. Atrial arrhythmias, including atrial fibrillation, atrial flutter, atrial tachycardia, and premature atrial contractions, are common observations in patients with CHD. The Framingham study reported that patients with CHD had a significantly higher risk of developing atrial fibrillation (2).

Antitumor drugs have significantly reduced morbidity and mortality in cancer patients (3). However, heart-related complications and death caused by these drugs have become another major challenge. Chemotherapy drugs can lead to changes in the electrocardiogram, tachycardia, and bradycardia (4, 5). However, there are limited studies exploring the impact of antitumor drugs on arrhythmia rates in patients with both malignant tumors and CHD. This study collected 109 patients with malignant cancer for the investigation of whether anti-tumor treatment could be associated with arrhythmia and the relevant potential risk factors. Our study reported the occurrence of atrial arrhythmias predominantly happened in cancer patients with CHD while undergoing antitumor treatment. Further analysis recognized the independent risk factors of smoking, ischemic heart disease, and EF < 50% in patients receiving anti-cancer treatment.

## Patients and methods

### Patient information

From January 2023 to January 2024, a total of 109 patients with lung cancer (51/109), liver cancer (45/109) and colon cancer (14/109) undergoing antitumor treatment at Zhejiang Hospital were selected for analysis. CHD was defined according to the diagnosis recorded in the hospital electronic medical record and confirmed by a cardiologist, based on available invasive or non-invasive assessments (such as coronary angiography, CT angiography, or stress imaging). Patients with only self-reported or unverified CHD were not counted as CHD patients. Those tumor patients with no CHD history were selected as the control group. Clinical data on baseline medication, tumor staging, chemotherapy regimen, laboratory tests, and echocardiography were collected from the medical records. This study complied with medical ethics standards and was approved by the ethics committee of our working institute. The purpose of the procedures was informed to the patients with receiving the signed consent of the patient or their family.

The inclusion criteria were: 1) diagnosed with malignant tumors; 2) availability of complete clinical data, including age, tumor TNM staging, pathological type, treatment history, smoking and drinking habits, etc.; 3) ECG recordings before and after each chemotherapy cycle.

The exclusion criteria were: 1) age under 18; 2) any existing arrhythmia recorded on ECG before antitumor treatment or a history of arrhythmias.

### Following-up assessment

During follow-up, all patients underwent 12-lead ECGs lasting more than 15 seconds, before and after each chemotherapy cycle. Atrial arrhythmias were defined as new-onset atrial fibrillation (AF), atrial flutter, atrial tachycardia, or premature atrial contractions (PACs). PACs were recorded as present when identified on ECG. The primary endpoint was the occurrence of new atrial arrhythmias, regardless of whether the arrhythmia resolved during follow-up. Echocardiography was conducted based on the clinical situation.

### Statistical analysis

All data were analyzed using SPSS software. Measured data were presented as mean ± standard deviation. Data in terms of case numbers were shown in cases and percentages, with P < 0. 05 considered as statistically significant.

## Results

### General data comparison in patients

In our study, we included a total of 109 patients. Among them, 44 patients had coronary heart disease (CHD) while the rest (65 patients) reported no CHD history. We observed 13 out of 109 cases (11.9%) of atrial arrhythmias occurred. The incidence of atrial arrhythmias in the coronary heart disease (CHD) group was significantly higher than that in the non-CHD group (27.27% vs 1.54%, P < 0.001) (**Table 1**). Amongst the 13 patients with atrial arrhythmias, 5 showed atrial fibrillation (38.46%), and 8 had premature atrial contractions (61.54%). No case of atrial flutter or sustained atrial tachycardia was observed (**Table 2**).

**Table 1:** Clinical characteristics of the cohort population.

Characteristics	Groups		Statistics
	CHD (n = 44)	Non-CHD (n = 65)	P value
Age, years	69.77 ± 8.54	61.32 ± 11.26	< 0.001
Female, n (%)	21 (47.72%)	32 (49.23%)	0.967
Hypertension, n (%)	27 (61.36%)	15 (23.08%)	< 0.001
Smoking, n (%)	19 (43.18%)	18 (27.69%)	0.142
Drinking, n (%)	11 (25%)	10 (15.38%)	0.317
Hyperlipidemia, n (%)	4 (9.09%)	4 (6.15%)	0.839
Diabetes, n (%)	10 (22.73%)	10 (15.38)	0.472
Cancer Stage (I + II), n (%)	12 (27.27%)	11 (16.92%)	0.289
Metastasis, n (%)	32 (72.73)	54 (83.08%)	0.289
BMI baseline, mean ± SD	22.74 ± 2.80	22.22 ± 2.16	0.30
Left ventricular EF	63.00 ± 9.31	67.17 ± 7.17	0.01
LVIDd (mm), mean ± SD	4.94 ± 0.71	4.75 ± 0.46	0.083
RBC (10 (6)/L), mean ± SD	4.03 ± 0.69	4.04 ± 0.67	0.9
Platelet (K/μL), mean ± SD	226.52 ± 67.91	212.05 ± 81.53	0.334
Creatinine (μM), mean ± SD	67.36 ± 20.74	68.05 ± 19.61	0.86
Atrial arrhythmias, n (%)	12 (27.27%)	1 (1.54%)	< 0.001

BMI, body mass index; EF, Ejection fraction; LVEDd, left ventricular end diastolic diameter; mm, millimeter; RBC, red blood cell.

**Table 2:** Types of arrhythmias.

Type of arrhythmias	Number (%)
Atrial fibrillation	5 (38.46%)
Premature atrial contractions	8 (61.54%)
Atrial tachycardia	0
Atrial flutter	0

In addition, the average age in the coronary heart disease group was significantly higher than that in the non-coronary heart disease group (P < 0.001), and the proportion of hypertension was also significantly higher in the coronary heart disease group (P < 0.001). The left ventricular ejection fraction was significantly lower in the coronary heart disease group (P = 0.01) (**Table 1**).

### Univariate logistic regression analysis

Furthermore, we performed univariate logistic regression analysis for the following variables: age (> 60 years), diabetes, immunotherapy treatment, anthracycline treatment, cancer stage, metastasis, body mass index, smoking, alcohol consumption, hypertension, hyperlipidemia, admission serum creatinine, ischemic heart disease, and medication use such as aspirin. The results indicated that age (P = 0.049), ischemic heart disease (P = 0.009), left ventricular ejection fraction (EF) <50% (P = 0.024), statins use (P = 0.033), β-blockers (P = 0.011), and aspirin (P = 0.019) were identified as risk factors for atrial arrhythmias following anti-cancer treatment in cancer patients (**Table 3**).

**Table 3:** Univariate logistic regression analysis of risk factors for arrhythmias.

Risk factor	Odds ratio	P value
Age (> 60 yr)	1.067 (1.002 – 1.137)	0.044
Ischemic Heart Disease	24.00 (2.987 – 192.812)	0.003
EF < 50%	17.273 (1.446 – 206.318)	0.024
Smoking	5.464 (1.554 – 19.213)	0.008
Statins	3.714 (1.113 – 12.392)	0.033
Beta-blockers	5.375 (1.472 – 19.626)	0.011
Aspirin	4.167 (1.264 – 13.737)	0.019

### Multivariate logistic regression analysis

Moreover, we also performed multivariate logistic regression analysis to understand whether incorporating age, smoking, alcohol consumption, hypertension, hyperlipidemia, ischemic heart disease, and treatment of statins, β-blockers or aspirin were recognized as the independent risk factor. The results showed that smoking (P = 0.009), ischemic heart disease (P = 0.006), and EF < 50% (P = 0.014) were independent risk factors for atrial arrhythmias following anti-cancer treatment in cancer patients (**Table 4**).

**Table 4:** Multivariate logistic regression analysis for arrhythmias.

Risk factor	Odds ratio	95% lower	95% upper	P value
Smoking	9.182	1.721	49.001	0.009
Ischemic Heart Disease	23.280	2.471	221.191	0/006
EF < 50%	85.076	2.461	2941.272	0.014

## Discussion

Cancer is one of the leading causes of death worldwide, meanwhile cardiovascular disease is the second most common cause of death (7). Arrhythmias that occur after antitumor treatment are potential side effects, due to which cancer patients are at increased risk of cardiovascular disease and mortality.

Atrial fibrillation (AF) is a common arrhythmia after antitumor treatment, and a meta-analysis of 191 clinical studies showed that the incidence of atrial fibrillation after antitumor drug therapy was 0.26 – 4.9% per year, and the most common antitumor drugs that caused AF were ibrutinib, clofarabine, and ponatinib (8). Another study used the World Health Organization (WHO) database Vigibase to identify 19 antitumor drugs that are closely related to AF (9).

Tyrosine kinase inhibitors (TKIs) displays broad effects on the myocardium and associated vasculature, thus promoting cardiotoxicity (6). Animal experiments in mice showed that left atrial fibrosis was significantly higher than that of the control group after 4 weeks of ibrutinib feeding, and atrial fibrillation was more likely to occur through esophageal electrical stimulation (10). TKI-mediated atrial remodeling is associated with oxidative stress, Ca^2+^ dysregulation, and inflammatory response (10, 11).

Immunomodulatory agents and immunotherapy inhibitors (IMiD) harness various components of the immune system and tumor microenvironment to inhibit cancer progression. Analysis using data from the U.S. Food and Drug Administration’s Adverse Event Reporting System (FAERS) database showed that atrial fibrillation was the most common arrhythmia associated with immune checkpoint therapy (12). In an observational study of 90 patients receiving chimeric antigen receptor T-cell (CAR-T) therapy, the plasma levels of interleukin-6 (IL-6) and IL-15 in patients with new-onset fibrillation events during treatment were significantly higher than those in the group without AF, which may be associated with the development of AF (13).

Anthracyclines appear to play a crucial role in the development of cardiotoxicity by inducing oxidative stress caused by nitric oxide synthase production of reactive oxygen species (ROS) and reactive nitrogen (14). In a study of 249 lymphoma patients treated with anthracyclines, 6% of patients had new-onset AF after chemotherapy during follow-up, and the incidence of acute heart failure was significantly increased in patients with new-onset AF (15).

Coronary artery disease is the most common cardiovascular disease (16), while AF is the most common arrhythmia (17). In population-based studies, the prevalence of angina increases with age. Among women aged 45–64 years, it rises from 5% to 7%, and among women aged 65–84 years, it rises from 10% to 12%. For men, the prevalence ranges from 4 – 7% in the 45–64 age group to 12 – 14% in the 65–84 age group (18). A study tracking 3,983 male Air Force recruits over 44 years found that the risk of atrial fibrillation increased with the occurrence of myocardial infarction (relative risk = 3.62) and angina (relative risk = 2.84). The relative risk of atrial fibrillation was strongest during ischemic heart disease events and diminished over time (19).

The results of this study involved patients with three types of malignant tumors, including lung cancer (51/109), colon cancer (14/109) and liver cancer (44/109), undergoing different anti-tumor treatment, including alkylating agents, kinase inhibitors, and immune checkpoint inhibitor. These patients combined with coronary heart disease (CHD) were more likely to develop atrial arrhythmias after receiving anti-tumor treatment. The incidence of atrial arrhythmias in the CHD group after anti-tumor treatment was significantly higher than that in the non-CHD group. Traditional risk factors for atrial fibrillation include ischemic heart disease, age, alcohol consumption, etc. (20, 21) In our study, multivariate logistic regression analysis indicated that a history of ischemic heart disease, smoking, and reduced left ventricular systolic function were independent risk factors for the development of atrial arrhythmias in cancer patients after receiving anti-tumor treatment. This is not only to confirm the traditional findings from previous studies, but also to identify new risks within the population of cancer patients undergoing therapies.

However, this study also holds several limitations. First, this study was conducted at a single center, which may restrict the generalizability of the current conclusions and thus limit the applicability of the current findings across diverse populations and treatment settings. In addition, our study included patients with different types of cancers, undergoing various anti-tumor treatment, however, due to the limited number of recruited patients and low occurrence of atrial arrhythmias, the analysis did not differentiate arrhythmia risk according to specific cancer types or antitumor drugs, as the limited sample size prevented meaningful subgroup analyses. Third, the follow-up period was limited to the duration of chemotherapy cycles within one year, which may have missed late-onset arrhythmias. Furthermore, spot ECG before and after each treatment cycle is a routine clinical workflow, which however could constrain the detection of transient arrhythmia occurrence.

The limited number of patients was mainly due to the single-center design, strict inclusion criteria, and the specific study scope focusing on atrial arrhythmias in cancer patients receiving antitumor therapy. Additionally, only patients with complete ECG data before and after each chemotherapy cycle were included, and the coexistence of both malignant tumors and confirmed coronary heart disease was relatively uncommon, further constraining the sample size. Future studies involving longer follow-up, multi-center collaboration for larger patient cohorts, and continuous or long-term ECG methods to detect the transient arrhythmias, are needed to further validate these results and to assess potential variations in arrhythmia risk across different cancer subtypes and treatment regimens. We also acknowledge that the inclusion of PACs, which may limit clinical impact compared with AF, could overestimate the overall incidence of atrial arrhythmias. However, PACs still reflected early atrial electrical instability induced by antitumor treatment. Future prospective studies using continuous ECG or Holter monitoring are beneficial for quantifying PAC burden and better defining their clinical relevance.

Overall, our findings of identifying ischemic heart disease, smoking history, and reduced left ventricular systolic function as independent risk factors for atrial arrhythmias implied that cancer patients with CHD should be undertaken more comprehensive assessment before initiating anti-tumor therapy. High-risk individuals may benefit from enhanced cardiac monitoring during the treatment. The study also highlights the collaboration between oncologists and cardiologists to ensure they safety and efficacy of planned treatment contributing to more personalized, risk-informed decision-making in the management of cancer patients.

## Conclusion

In summary, patients with malignant tumors and coexisting coronary heart disease are at an increased risk of developing atrial arrhythmias during antitumor treatment. Smoking, ischemic heart disease, and reduced left ventricular ejection fraction were identified as independent risk factors. These findings highlight the importance of comprehensive cardiovascular evaluation and enhanced cardiac monitoring before and during cancer therapy.

Future multi-center collaborative studies with larger sample sizes and stratified analyses based on cancer types and specific therapeutic agents can validate and refine these observations, contributing to more personalized and risk-informed cardio-oncology care.

## Data Availability

The raw data supporting the conclusions of this article will be made available by the authors, without undue reservation.
